# Migraine and epilepsy: what value today?

**DOI:** 10.1186/1129-2377-16-S1-A44

**Published:** 2015-09-28

**Authors:** Cinzia Costa, Paolo Prontera, Stefano Caproni, Letizia M Cupini, Paola Sarchielli, Paolo Calabresi

**Affiliations:** Clinica Neurologica, Università di Perugia, Ospedale S. Maria della Misericordia, Perugia, Italy; Sezione di Genetica Medica, Dipartimento di Medicina Clinica e Sperimentale, Azienda Ospedaliera e Università di Perugia, Perugia, Italy; Centro Cefalee, UOC Neurologia, Ospedale S. Eugenio, Rome, Italy; Fondazione Santa Lucia, IRCCS, Rome, Italy

More than 100 years of investigation have found that seizures and migraine co-occur in some affected individuals and families, and there is much evidence that these diseases share genetic susceptibility. In particular, in a recent review, the prevalence of migraine in epileptic patients was about 12%[1]. On the other hand, epilepsy exhibits a prevalence of about 6% in migraineurs[2]. Moreover, epilepsy has also been reported in patients with familial hemiplegic migraine (FHM). Although few studies showed a prevalence of epilepsy of about 7% in FHM, currently no conclusive data are available.

Certainly, epilepsy and migraine share common characteristics that the underlying pathophysiology relates to alterations in ion channels or ion transporters. In these episodic functional diseases, in which susceptible brain regions are hyperexcitable, the attacks begin with hypersynchronous neuronal firing[3]. In epilepsy, α-amino-3-hydroxy-5-methyl-4-isoxazolepropionic acid (AMPA) receptors play a predominant role in mediating the generation and spread of seizure activity whereas in migraine mainly N-methyl-D-aspartate (NMDA) receptors are involved in triggering CSD. However, the nature of the ionic conductance changes leading to the massive but transitory neuronal depolarization underlying CSD has not yet been define[4].

Genetically determined dysfunction of ion transporters seems to point at a common underlying mechanism for both paroxysmal disorders. In the last two decades the mutations in the ion transportation genes *CACNA1A*, *ATP1A2* and *SCN1A* have been identified for FHM. Conversely, only a few *CACNA1A* and *ATP1A2* mutations have been reported in patients with sporadic hemiplegic migraine (SHM). These cases can be caused by a *de novo* mutation or by inheritance of a gene mutation from asymptomatic carrier and are usually characterized by early-onset, severe and complex disorders.

Certainly, genetic analysis can provide greater insight into the potential causes of both disorders and it could contribute to better treatment choices.
